# Association between *col1a2* Polymorphism and the Occurrence of Pelvic Organ Prolapse in Brazilian Women

**DOI:** 10.1055/s-0038-1676599

**Published:** 2019-01

**Authors:** Josyandra Paula de Freitas Rosa, Raphael Federicci Haddad, Fabiana Garcia Reis Maeda, Ricardo Peres Souto, Cesar Eduardo Fernandes, Emerson de Oliveira

**Affiliations:** 1 Faculdade de Medicina do ABC, Santo André, SP, Brazil

**Keywords:** procollagen type I alpha (α) 2 gene, polymorphism, pelvic organ prolapse, gene pró-colágeno tipo I alfa (α) 2, polimorfismo, prolapso de órgãos pélvicos

## Abstract

**Objective** To evaluate the rs42524 polymorphism of the procollagen type I alpha (α) 2 (*COL1A2*) gene as a factor related to the development of pelvic organ prolapse (POP) in Brazilian women.

**Methods** The present study involved 112 women with POP stages III and IV (case group) and 180 women with POP stages zero and I (control group). Other clinical data were obtained by interviewing the patients about their medical history, and blood was also collected from the volunteers for the extraction of genomic DNA. The promoter region of the *COL1A2* gene containing the rs42524 polymorphism was amplified, and the discrimination between the G and C variants was performed by digestion of the polymerase chain reaction (PCR) products with the MspA1I enzyme followed by agarose gel electrophoresis analysis.

**Results** A total of 292 women were analyzed. In the case group, 71 had the G/G genotype, 33 had the G/C genotype, and 7 had the C/C genotype. In turn, the ratio in the control group was 117 G/G, 51 G/C, and 11 C/C. There were no significant differences between the groups.

**Conclusion** Our data did not show an association between the *COL1A2* polymorphism and the occurrence of POP.

## Abstract (pt)

**Resumo:**

**Objetivo** Avaliar o polimorfismo rs42524 do gene pró-colágeno tipo I alfa (α) 2 (*COL1A2*) como fator relacionado ao desenvolvimento de prolapso de órgãos pélvicos (POP) em mulheres brasileiras.

**Métodos** O estudo envolveu 112 mulheres com POP nos estádios III e IV (grupo caso) e 180 mulheres com POP nos estádios zero e I (grupo controle). Outros dados clínicos foram obtidos por meio de entrevistas com as pacientes sobre seu histórico médico, e o sangue das voluntárias também foi coletado para extração de DNA genômico. A região promotora do gene *COL1A2* contendo o polimorfismo rs42524 foi amplificada, e a discriminação entre as variantes G e C foi realizada por digestão dos produtos de reação em cadeia da polimerase (RCP) com a enzima MspA1I, seguida de análise por eletroforese em gel de agarose.

**Resultados** Foram analisadas 292 mulheres. No grupo caso, 71 tinham o genótipo G/G, 33 tinham o genótipo G/C, e 7 tinham o genótipo C/C. Por sua vez, a relação no grupo controle foi de 117 G/G, 51 G/C e 11 C/C. Não houve diferenças significativas entre os grupos.

**Conclusão** Nossos dados não mostraram associação do polimorfismo do gene *COL1A2* com a ocorrência de POP.

## Introduction

Pelvic organ prolapse (POP) is a pathological condition characterized by the displacement of pelvic viscera in the caudal direction, towards the genital hiatus. The standardization of its symptoms was updated in 2016 by the International Continence Society (ICS) and by the International Urogynecology Association (IUGA). They considered the following complaints to be related to POP: vaginal bulging, pelvic or suprapubic pressure, bleeding, discharge and infections related to ulceration, need for manual maneuvers to facilitate defecation or urination, and pain in the sacral spine region.^1^

The prevalence of POP varies between 8 and 41%, contributing substantially to the reduction of the quality of life of the patients, as they experience physical symptoms, but also to problems related to general health, personal relationships, and sexual function.^2^ In addition, POP is the main reason for surgery in aging women.^3^

The etiology of POP is considered multifactorial, and several risk factors, such as advanced age, pregnancy, obesity, neuropathies, ethnicity, hysterectomy, instrumental delivery, and menopause, have been identified. Recent evidence suggests the existence of a genetic component with a 3.2- to 2.4-fold increased risk for the mothers and sisters of affected women, respectively. The high prevalence of POP in patients with type I and III collagen disorders, such as Ehlers-Danlos and Marfan syndromes, corroborates the importance of studying the genetic background of patients with the disease.^3^ ^4^

Older age and parity are the most significant etiological factors, but they do not fully explain the origin and progression of pelvic floor dysfunction in all women, since POP has been observed in nulliparous women and has been absent in many multiparous women.^5^ The main mechanism of pelvic floor dysfunction is the weakening of the structures that support the pelvic organs: connective tissue in the form of ligaments and endopelvic fascia, and the levator ani muscles.^6^

The main protein structure of connective tissues is type I collagen, a heterotrimer with two α-1 chains and one α-2 chain encoded by the procollagen type I alpha (α) 1 (*COL1A1*) and procollagen type I alpha (α) 2 (*COL1A2*) genes, respectively. This protein is physiologically important to support the pelvic floor structures and to confer mechanical stability to the genitourinary tract.^7^ The vaginal fascia and its ligaments are formed predominantly by collagen type I and III, which enable the accommodation of the structures in cases such as sudden increased abdominal pressure and the passage of a fetus.

In the case of collagen changes, the pelvis becomes more susceptible to genital prolapse, as the fascia and its ligaments are put under stress during periods of increased intra-abdominal pressure. Thus, it is believed that an abnormal connective tissue metabolism may be associated with this gynecological condition.^7^

The *COL1A2* gene is located on the chromosome 7q22.1.^8^ It contains 52 exons and non-coding portions (38 kb in size).^9^ It encodes the procollagen alpha 2 chain, a component of the collagen type I molecule.^10^ ^11^

Each α-chain contains terminal propeptides in the C-terminus (carboxy) and in the N-terminus (amino), and a core domain composed of 338 Gly-X-Y repeats, where X and Y exclude cysteine and tryptophan, and are often proline and hydroxyproline, respectively. Glycine, as the smallest amino acid, is the only residue capable of occupying the axial position of the triple helix, so that any change from a glycine residue will entail disruption of the helical structure.^12^ ^13^

Genetic polymorphism is the presence of variation in the DNA sequence found at a frequency of > 1% of the population. A single-nucleotide polymorphism (SNP) is a site in the DNA where a single base pair or nucleotide varies from person to person. As genetic markers, SNPs can be used to track inheritance patterns of chromosomal regions from generation to generation. Although most of the polymorphisms are inactive, some may influence the promoter activity or the conformation of DNA and pre-mRNA, which may result in a change in the amino acid sequence and in the protein function and, therefore, in the phenotypic expression.^6^ The frequency of SNPs can be measured and has been associated with different risks of development of diseases.^14^

Mutations that affect the *COL1A2* gene reduce the biosynthesis of type I collagen and, consequently, could be involved in osteogenesis imperfecta.^15^ According to the literature, a polymorphism of the *COL1A2* is associated with vascular disease and osteoporotic fractures.^16^ ^17^ ^18^ A recent meta-analysis suggests that *COL1A2* rs42524 is a significant risk factor for intracranial aneurysm susceptibility, with an especially strong effect in Asian people.^19^ However, in our review, it was not possible to find studies evaluating the relationship of this polymorphism with POP.

Given the aging of the Brazilian population, which is estimated to be predominantly composed of adults and the elderly, according to the Brazilian Institute of Geography and Statistics (IBGE, in the Portuguese acronym), and considering the high morbidity of this gynecological condition and the high costs of its treatment, it can be concluded that POP is a highly relevant problem for the global public health.^20^ ^21^

Thus, an important goal on the agenda of the urogynecological scientific community is to develop tools and markers capable of predicting which women will develop POP, so that they receive adequate follow-up, particularly in terms of obstetric care. For this purpose, the identification of genetic polymorphisms, more or less frequent in women with POP compared with the general population, can be the basis for an early disease risk assessment and is therefore extremely important.

Therefore, the objective of the present work is to verify the association between *COL1A2* polymorphisms (rs42524) with POP.

## Methods

This is a cohort study with a total of 292 postmenopausal women. The patients were treated at the Department of Urogynecology and Vaginal Surgery of the Department of Gynecology and Obstetrics of a School of Medicine. To ensure the rights and duties of the scientific community, of the study population and of the State, the present study complied with the guidelines of Resolution 196/96 of the National Health Council (CNS, in the Portuguese acronym) and, therefore, was previously submitted to evaluation and approval by the Research Ethics Committee of the Faculdade de Medicina do ABC (FMABC, in the Portuguese acronym) under the number 554.670/2014. All of the patients were informed about the study and signed a consent form for participation.

The inclusion criteria were: (1) diagnosis, by physical examination, of POP stages III and IV in the case group, and of POP stages zero and I in the control group; (2) a history compatible with postmenopause (absence of menstrual bleeding for at least a year); and (3) no hormone therapy in the previous 6 months for both groups.

The exclusion criteria were: (1) lack of permission from the patient to perform blood collection after having been informed about the study in both groups; and (2) patients who underwent any type of prior vaginal surgery in the control group.

In the medical interview, the following data were collected: age, ethnicity, body mass index (BMI), parity, place of birth and delivery route, obstetric interventions (analgesia and episiotomy), weight of the heaviest newborn, previous diseases (diabetes, hypertension, dyslipidemia, chronic cough, and constipation), life habits (physical activity with high physical exertion, and smoking), and previous hysterectomy.

All of the women were weighed and measured for the calculation of their BMI, followed by a gynecological examination, in which POP-Q staging was performed for the quantification of genital prolapse.^22^

**Fig. 1:**
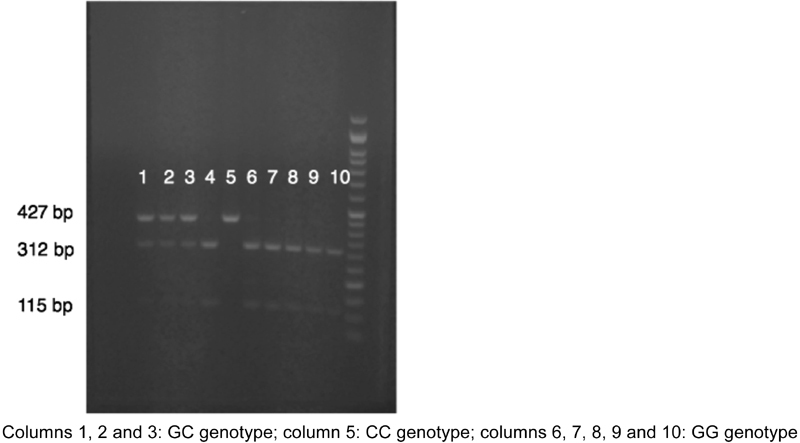
Representative results for rs42524 genotyping of the *COL1A2* gene.

For the extraction of genomic DNA, the illustra blood genomicPrep Mini Spin Kit (GE Healthcare Life Sciences, Chicago, IL, USA) was used, following the instructions of the manufacturer. The amplification reaction was performed in 20 μL using an adequately diluted PCR Master Mix reagent (Promega Corporation, Madison, WI, USA) and the primers described by Liu et al (2012): 23 5′-TACCTGAGGCTTTGAGAC-3′ and 5′-GAAAATATAGAGTTTCCAGAG-3′. The amplification protocol was as follows: initial incubation at 94° C for 5 minutes followed by 45 cycles of 3 temperatures (94° C for 30 seconds, 49° C for 30 seconds, 72° C for 60 seconds) and a final incubation at 72° C for 10 minutes. The samples were then kept at 10° C until the electrophoresis was performed. The polymerase chain reaction (PCR) products were digested with the restriction endonuclease MspA1I for 16 hours and visualized on agarose gel stained with ethidium bromide. The genotypes were determined by the observed pattern of the digestion bands: a single 427 base pair (bp) for homozygous CC (mutant homozygous genotype); two bands of 312 and 115 bp for homozygous GG (wild type genotype), and three bands of 427, 312 and 115 bp for heterozygous GC (mutant heterozygous genotype) (Fig. 1).

The normality of the quantitative data was verified using the Shapiro-Wilk test. The qualitative variables were compared using the chi-squared and the Fisher exact tests. An unpaired t-test was used to compare the quantitative variables. The data were analyzed using GraphPad Prism 6.0 (GraphPad Software, La Jolla, CA, USA) and IBM SPSS Statistics for Windows, Version 23.0 (IBM Corp., Armonk, NY, USA). After the stratification of the groups, the influence of the clinical characteristics on the risk of POP was estimated using odds ratios (ORs) obtained from the binary logistic regression model. The adopted significance level was 5% (*p* < 0.05), and the adopted confidence interval (CI) was 95%.

## Results

A total of 180 women with stage zero or I (control group) and 112 women with stage III or IV (case group) were selected for analysis. The groups were significantly different in terms of age, parity, number of pregnancies, and number of vaginal deliveries and home deliveries (Table 1).

**Table 1:** Analysis of the clinical characteristics of women with and without pelvic organ prolapse

Variables	Case group (*n* = 112) mean or %	Control group (*n* = 180) mean or %	*p*-value*
Age, mean (years old)	68.4	57.8	< 0.0001^a^
White	69.90%	64.80%	0.422^b^
Non-White	30.10%	35.20%	
Body mass index (kg/m^2^)	28.8	28.9	0.874^a^
Age of menopause (years old)	48.8	46.6	0.07^a^
Hormonal therapy	10.70%	18.10%	0.09^b^
Smoking	13.10%	20.10%	0.15^b^
Arterial hypertension	57.80%	49.40%	0.186^b^
Diabetes mellitus	24.50%	23.70%	0.888^b^
Dyslipidemia	25.40%	24.70%	0.889^b^
Chronic cough	1.80%	6.80%	0.08^b^
Constipation	14.30%	10.40%	0.35^b^
Pregnancy	5.6	3.5	< 0.0001^a^
Parity	4.8	2.9	< 0.0001^a^
Vaginal delivery	4.1	2.3	< 0.0001^a^
C-section	0.08	0.12	0.377^a^
Weight of the heaviest newborn (g)	3,516	3,059	0.147^a^
Episiotomy	8.30%	9.20%	> 0.99^b^
Labor analgesia	3.70%	4.80%	0.768^b^
Home birth	25.90%	3.05%	< 0.0001^b^
Hysterectomy	15.20%	15.60%	> 0.99^b^
Exaggerated physical exercise	22.50%	14.10%	0.077^b^

a Unpaired t test;

b Fisher exact test.

The independent risk factors for the development of POP were determined by calculating the OR using the logistic regression model and are shown in Table 2. Following the adjusted OR analysis, we found a statistical significance only for the variables age ≥ 51 years old and home delivery.

**Table 2:** Logistic regression of factors associated with the risk of pelvic organ prolapse

Factors	Crude OR (95% CI)	*p*-value	Adjusted OR (95% CI)
Age ≥ 51	15.57 (4.73–51.2)	< 0.0001	11.89 (3.53–40)
Pregnancy ≥ 3	2.02 (1.24–3.28)	0.004	0.656 (0.283–1.51)
Vaginal delivery ≥ 3	3.12 (1.86–5.23)	< 0.0001	1.91 (0.7–5.22)
Parity ≥ 3	2.64 (1.62–4.31)	< 0.0001	2.01 (0.665–6.1)
Home birth	11.1 (4.14–29.7)	< 0.0001	9.645 (3.35–27.7)

Abbreviations: CI, confidence interval; OR, odds ratio.

There was a tendency for the Hardy-Weinberg equilibrium in both groups regarding the frequency of the genotypes studied (*p* = 0.046). We did not have DNA amplification in one patient in the case group and in another in the control group, totaling two cases of non-amplified DNA. Conversely, the presence or absence of *COL1A2* gene polymorphisms was not associated with the presence of genital prolapse (Table 3).

**Table 3:** Distribution of the frequencies of *COL1A2* genotypes between cases and controls

*COL1A2* genotypes	Case group (*n* = 111)	Control group (*n* = 179)	*p*-value
GG	71 (63.9%)	117 (65.3%)	0.9705^a^
GC	33 (29.7%)	51 (28.5%)	
CC	7 (6.4%)	11 (6.2%)	
*COL1A2* aggregated genotypes			
GG	71 (63.9%)	117 (65.3%)	0.7105^b^
GC + CC	40 (36.1%)	62 (34.7%)	

Abbreviations: CC, mutant homozygous genotype; GC, mutant heterozygous genotype; GG, wild type genotype.

a Chi-squared test;

b Fisher exact test.

## Discussion

The identification of patients who are susceptible to the development of POP may lead to preventive treatment. Several genetic studies have already been conducted, revealing different candidate genes and chromosomal loci that are associated with the risk of POP.

Pelvic organ prolapse affects negatively the quality of life of women, especially after the age of 50 years old; among these women, approximately 10% will require surgery by the age of 80 years old. The etiology of POP is multifactorial; of late, genetic factors have been extensively studied.^10^ ^11^

Our results demonstrated a higher, albeit not statistically significant, prevalence of the *COLIA2* polymorphism among patients with advanced POP. Genetic polymorphisms in the genes encoding α-1 and α-2 chains of type I collagen and its influence on POP were studied in the world literature. A Brazilian study demonstrated that there were no differences in the prevalence of the GT and TT genotypes of the *COL1A1* gene between the groups even when we grouped patients with at least one polymorphic allele (GT and TT) and compared them with patients without the polymorphic allele (GG). Moreover, the *COL1A1* Sp1-binding site was not significantly associated with genital prolapse among our study subjects.^24^ Another study of particular interest was the finding that the only case of polymorphic homozygosity (TT) of the *COL1A1* gene was found in the control group (that is, women without POP), suggesting that the GT genotype has a stronger association with POP than the TT genotype.^25^

It has recently been shown that a common coding polymorphism (rs42524) in the *COL1A2* gene, which replaces alanine for proline at position Y of the helical region of alpha 2 (I) collagen, could be a genetic risk factor for aneurysms.^26^ This study was followed by several others studying the association of this polymorphism with osteoporosis and vascular disease. Lindahl et al (2009)^18^ showed that the heterozygote genotype had an increased risk of stroke, myocardial infarction, and lower bone mineral density. In turn, Majchrzycki et al (2017)^17^ found that the *COL1A2* gene polymorphism may be a genetic risk factor related to the development of osteoporosis; and, lastly, Meng et al (2017)^16^ observed in a meta-analysis that rs42524 in the *COL1A2* gene was associated with a significant increase in the risk of intracranial aneurysms in Japanese patients (allelic model: OR = 1.94; 95% CI: 1.03–3.64; *p* = 0.04).

In our study, we investigated the possible association of the rs42524 polymorphism of the *COL1A2* gene with the occurrence of POP. This is a pioneer work in the literature, and it failed to demonstrate the association of this polymorphism with POP.

We acknowledge that among the limitations of our work is the fact that our samples were not completely homogeneous in terms of some clinical characteristics, likely because advanced degrees of prolapse are more frequent in older women with more gestations and deliveries, who, in this case, correspond to those of the study group. Other limitations of our study are the fact that it only evaluated a single polymorphim and gene, as well as the miscegeneation of the Brazilian population, as well as the lack of equilibrium of Hardy-Weinberg. Regarding the latter, perhaps the small number of patients may be responsible for the absence of the genic equilibrium. In order to ensure that the genotyping was adequate, the PCR was always performed by the same three observers, and their consensus was recorded.

We believe that further studies that seek to establish genetic markers for POP are necessary and, therefore, the present study may be useful for future meta-analyses. We also think that we cannot extrapolate the conclusion for other populations based on these results. This study is specially important because the capacity to identify individuals at greater risk of developing POP through genetic screening could be useful in cases such as deciding the most appropriate delivery route for large fetuses.

Hopefully, focusing on the genetic susceptibility to POP will allow the stratification of risk for women who develop POP and thus establish strategies for its prevention and lifestyle changes. These interventions could reduce the need for corrective surgery and could improve the quality of life of women with the most severe stages of genital prolapse.

## Conclusion

In conclusion, our data did not demonstrate an association between the *COL1A2* polymorphism and the occurrence of POP.
